# NuSAP modulates the dynamics of kinetochore microtubules by attenuating MCAK depolymerisation activity

**DOI:** 10.1038/srep18773

**Published:** 2016-01-06

**Authors:** Chenyu Li, Yajun Zhang, Qiaoyun Yang, Fan Ye, Stella Ying Sun, Ee Sin Chen, Yih-Cherng Liou

**Affiliations:** 1 Department of Biological Sciences, Faculty of Science, National University of Singapore, 14 Science Drive 4, 117543, Republic of Singapore; 2 Department of Biochemistry, Yong Loo Lin School of Medicine, National University of Singapore, Singapore 117597, Republic of Singapore; 3 NUS Graduate School for Integrative Sciences and Engineering, National University of Singapore, Singapore 117573, Republic of Singapore

## Abstract

Nucleolar and spindle-associated protein (NuSAP) is a microtubule-associated protein that functions as a microtubule stabiliser. Depletion of NuSAP leads to severe mitotic defects, however the mechanism by which NuSAP regulates mitosis remains elusive. In this study, we identify the microtubule depolymeriser, mitotic centromere-associated kinesin (MCAK), as a novel binding partner of NuSAP. We show that NuSAP regulates the dynamics and depolymerisation activity of MCAK. Phosphorylation of MCAK by Aurora B kinase, a component of the chromosomal passenger complex, significantly enhances the interaction of NuSAP with MCAK and modulates the effects of NuSAP on the depolymerisation activity of MCAK. Our results reveal an underlying mechanism by which NuSAP controls kinetochore microtubule dynamics spatially and temporally by modulating the depolymerisation function of MCAK in an Aurora B kinase-dependent manner. Hence, this study provides new insights into the function of NuSAP in spindle formation during mitosis.

Kinetochore microtubules directly connect to kinetochores on sister chromatids to generate proper tension and ensure error-free chromosome separation^1^,^2^. During metaphase, the dynamics of kinetochore microtubules are tightly controlled by microtubule-associated proteins, motor proteins, and mitotic kinases to precisely align chromosomes at the metaphase plate^3^,^4^,^5^,^6^,^7^. Disruption of this process leads to chromosome instability, which is considered to be one of the main causes of carcinogenesis^8^,^9^,^10^.

Nucleolar spindle-associated protein (NuSAP) is a microtubule-associated protein that plays an important role in spindle assembly^11^,^12^. Previous studies showed that depletion of NuSAP in cells resulted in defective mitotic spindle formation, chromosome segregation, and cytokinesis^11^. NuSAP was identified as a microtubule stabiliser as a result of its ability to induce microtubule crosslinking, bundling, and attachment to chromosomes^13^,^14^. The protein levels of NuSAP are tightly regulated by anaphase-promoting complex/cyclosome (APC/C) during the cell cycle^15^,^16^, and high expression of NuSAP was observed in several types of cancers^17^,^18^,^19^,^20^,^21^,^22^. Although a number of studies have explored the role of NuSAP, its mechanism of action remains largely unknown.

Mitotic centromere-associated kinesin (MCAK) is a member of the kinesin-13 family^23^ and an important microtubule depolymeriser^24^,^25^,^26^. During mitosis, MCAK relocalises to the inner kinetochore region at metaphase^27^,^28^ where it is able to remove mis-connected kinetochore microtubules^27^,^28^,^29^. The depolymerisation activity of MCAK is tightly regulated though phosphorylation by Aurora B kinase, the catalytic subunit of the chromosomal passenger complex^30^. Aurora B, which is concentrated between sister chromatids from prometaphase to metaphase^31^,^32^, corrects imprecise attachment of kinetochore microtubules and regulates kinetochore microtubule dynamics to ensure accurate chromosome alignment^33^,^34^. However, it remains unclear whether additional regulators of MCAK exist during mitosis.

Here, using a combination of microscopy and biochemical techniques, we sought to identify potential binding partners of NuSAP in order to understand the mechanism by which NuSAP stabilises kinetochore microtubules. Our study provides new insights into the pivotal role of NuSAP in maintaining the fidelity of chromosome segregation during mitosis.

## Results

### NuSAP stabilises kinetochore microtubule during metaphase

**Figure 1:**
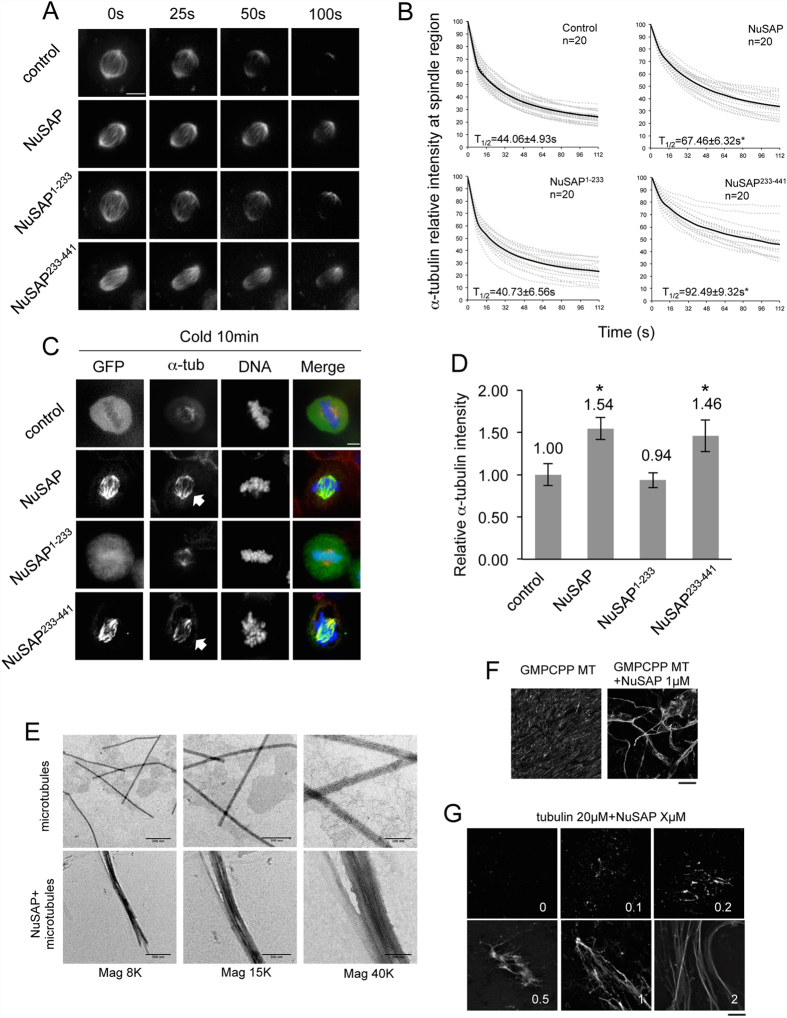
NuSAP stabilises kinetochore microtubules during metaphase. (**A**) Representative images of spindle microtubule signal loss in mCherry-α-tubulin stable metaphase HeLa cells and HeLa cells expressing GFP-NuSAP, GFP-NuSAP^1–233^, or GFP-NuSAP^233–441^ analysed by FLIP assay. Scale bar, 5 μm. (**B**) Normalised signal-decreasing curves of mCherry-α-tubulin signal intensity at the metaphase spindle region in FLIP assays. Dotted grey lines represent each individual measurement and black lines represent the mean value of each group. Turnover half-life was calculated by linear regression analysis. Data were collected from three independent experiments, and “n” indicates the total number of mitotic spindles analysed. Error bars represent ± SD. *p < 0.001. (**C**) Kinetochore microtubules in cold-treated metaphase HeLa cells expressing GFP-NuSAP, GFP-NuSAP^1–233^, GFP-NuSAP^233–441^, or GFP vector (control). Cells were stained with anti-α-tubulin antibody and DNA labelled with Hoechst 333342. Scale bar, 5 μm. Arrows indicate kinetochore bundles. (**D**) Bar chart representing average α-tubulin immunofluorescence intensity on metaphase spindles stained as C in cells expressing GFP-NuSAP, GFP-NuSAP^1–233^, GFP-NuSAP^233–441^, and GFP vector only (control). Data were collected across three independent experiments. The number of cells quantified was 37, 36, 35, and 39 for GFP, GFP-NuSAP, GFP-NuSAP^1–233^, and GFP-NuSAP^233–441^, respectively. Error bars represent ± SD. *p < 0.001. (**E**) Purified microtubules (1.5 μM) were incubated either alone or with 1 μM recombinant NuSAP for 10 min and fixed for electron microscopy. In the presence of NuSAP, microtubule bundles were detectable. (**F**) GMPCPP microtubules (2 μM) were incubated either alone or with 1 μM NuSAP for 10 min. Scale bar, 20 μm. (**G**) Different concentration of NuSAP was incubated with 20 μM tubulin for 10 min. Scale bar, 20 μm.

To study the function of NuSAP during metaphase, we constructed vectors expressing full-length NuSAP, the N-terminal domain (1–233 aa, NuSAP^1–233^), which includes the chromosome-binding domain, and the C-terminal domain (233–441 aa, NuSAP^233–441^), which contains the microtubule-binding domain (MTBD) (Fig. S1A). As expected, NuSAP and NuSAP^233–441^, but not NuSAP^1–233^, localised at the spindle microtubules during metaphase (Fig. S1B). HeLa cells overexpressing NuSAP or NuSAP^233–441^, but not NuSAP^1–233^, also retained more stable spindle microtubules than control cells after nocodazole treatment, which is known to depolymerise microtubules (Fig. S1C,D). To investigate the function of NuSAP in stabilising microtubules further, we performed a FLIP (fluorescence loss in photobleaching) assay in NuSAP-transfected HeLa cells that stably express mCherry-tagged α-tubulin. The half-lives (T_1/2_) of spindle microtubules in NuSAP- and NuSAP^233–441^-overexpressing cells were 67.46 ± 6.32 sec and 92.49 ± 9.32 sec, respectively, considerably longer than those of the control (44.06 ± 4.93 sec) and NuSAP^1–233^-transfected cells (40.73 ± 6.56 sec) (Fig. 1A,B). These results suggest that NuSAP functions as a microtubule stabiliser through its C-terminal microtubule-binding domain by decreasing microtubule turnover rate.

To further decipher the specific function of NuSAP at kinetochore microtubules during metaphase, we performed a cold treatment to selectively depolymerise interpolar microtubules but retain kinetochore microtubules^35^,^36^,^37^. Overexpression of NuSAP or NuSAP^233–441^ resulted in the formation of stable cold-resistant kinetochore microtubule bundles (Fig. 1C, indicated by arrows) with an increased fluorescent intensity of α-tubulin observed on metaphase spindles (Fig. 1D). To investigate the role of NuSAP in microtubule bundle formation further, microtubules were incubated with purified recombinant full-length NuSAP protein *in vitro*. We observed prominent formation of microtubule bundling in the presence of 1 μM NuSAP (Fig. 1E,F), which is consistent with a previous report^13^. NuSAP was also found to increase microtubule regrowth *in vitro* in a concentration-dependent manner (Fig. 1G), and to increase nucleation in Hela cells *in vivo* (Fig. S2A,B).

To examine kinetochore microtubule stability directly, HeLa cells were cold-treated with monastrol to remove interpolar microtubules from monopolar cells. Measurement of the kinetochore microtubule length from one kinetochore to the nearest centrosome^38^ revealed that the average kinetochore microtubule distance was significantly increased in NuSAP- (3.67 ± 0.31 μm) and NuSAP^233–441^-overexpressing cells (3.52 ± 0.24 μm), but not in NuSAP^1–233^-overexpressing cells (2.47 ± 0.23 μm) compared with control cells (2.41 ± 0.23 μm) (Fig. S2C,D). These results indicate that NuSAP stabilises kinetochore microtubules during metaphase.

### NuSAP associates with and modulates the microtubule destabilising activity of MCAK

**Figure 2:**
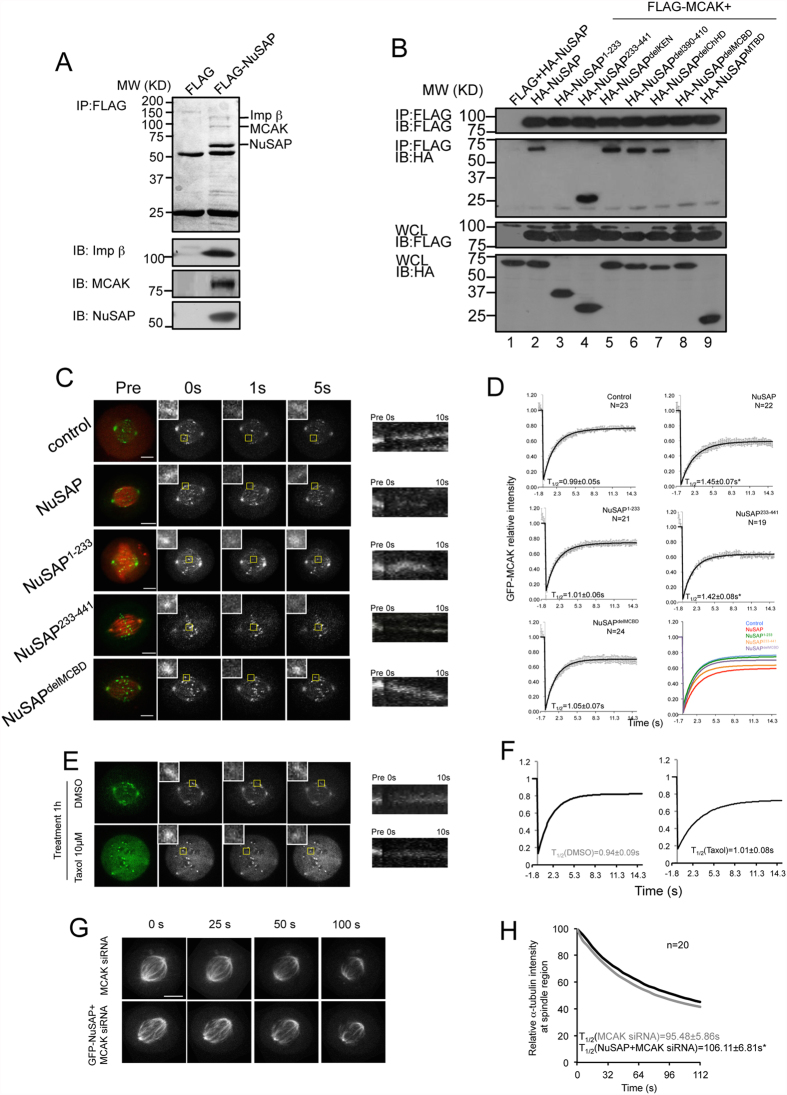
NuSAP binds with MCAK and modulates the dynamics of MCAK. (**A**) NuSAP immunoprecipitates contain MCAK and importin-β. FLAG and FLAG-NuSAP immunoprecipitates from HEK 293T cell lysates were analysed by Coomassie blue staining. The indicated protein bands were identified by mass spectrometry and confirmed by western blotting using anti-MCAK, anti-importin-β, and anti-NuSAP antibodies. (**B**) Identification of the MCAK-binding domain on NuSAP. Immunoprecipitated proteins were detected with the use of anti-HA and anti-FLAG antibodies. Blotting of proteins in whole cell lysates (WCL) are also shown as controls. (**C**) Representative images and kymographs representing MCAK dynamics at the kinetochore region in metaphase HeLa cells expressing mCherry-vector (control), mCherry-NuSAP, mCherry-NuSAP^1–233^, mCherry-NuSAP^233–441^, or mCherry-NuSAP^delMCBD^. Yellow squares represent the 1 × 1-μm photobleaching region. Kymographs were generated from the photobleaching kinetochore region. Scale bar, 5 μm. (**D**) Normalised signal recovery curves of FRAP assays. Solid lines represent the fit values of each group, and dots indicate the mean values. Turnover half-life was calculated using a single constrained exponential curve. Data were collected from three independent experiments and “n” indicates the number of mitotic spindles analysed. Error bars represent ± SD. *p < 0.001. (**E**) Representative images and kymographs of MCAK dynamics at kinetochore region in metaphase HeLa cells treated with DMSO or 10 μM Taxol for 1 hour. Scale bar, 5 μm. (**F**) Normalised signal recovery curves of FRAP assays. Error bars represent ± SD. *p < 0.001. (**G**) Representative images of spindle microtubule signal loss in MCAK siRNA transfected HeLa cells and HeLa cells expressing GFP-NuSAP with FLIP assays. Scale bar, 5 μm. (**H**) Normalised signal decreasing curves of mCherry-α-tubulin signal intensity at the metaphase spindle region in FLIP assays. Error bars represent ± SD. *p < 0.001.

We sought to identify the binding partners of NuSAP in order to understand the molecular mechanism by which NuSAP regulates the stability of microtubules. Proteomic analysis of proteins immunoprecipitated from HEK 293T cell lysates with FLAG-tagged NuSAP identified two proteins, importin subunit beta-1 and KIF2C (also known as MCAK). MCAK was previously identified as an important microtubule depolymeriser (Fig. 2A and Table S1) so we decided to study this interaction further. We delineated the MCAK-binding domains of NuSAP by performing further immunoprecipitation assays with a series of truncated mutants of NuSAP. Deletion of amino acids 433-441 from the C-terminal of NuSAP resulted in a marked abolishment of the interaction between NuSAP and MCAK, confirming that the region represented by amino acids 433–441 hosts the MCAK-binding domain (MCBD) of NuSAP (Figs 2B and S3A).

Since the dynamics of MCAK localisation are essential for its depolymerisation activity, we investigated the effect of NuSAP on MCAK dynamics by FRAP (Fluorescence Recovery After Photobleaching) assay. MCAK signal at the 1 × 1 μm kinetochore region was photobleached and analysed in HeLa cells overexpressing NuSAP or truncated NuSAP mutants (Fig. 2C). Kymographs generated from the 1 × 1 μm bleaching region, based on MCAK signal intensity recovery 10 sec after photobleaching, indicated that NuSAP and NuSAP^233–441^, but not NuSAP^1–233^ or NuSAP^delMCBD^, noticeably reduced the dynamics of MCAK (Fig. 2C, right panel). FRAP analyses were also performed to quantify the dynamics of MCAK, with normalised intensity fitted to a constrained exponential curve (Fig. 2D). The half-lives of MCAK were significantly lengthened in NuSAP- (1.45 ± 0.07 sec) and NuSAP^233–441^-overexpressing cells (1.42 ± 0.08 sec) compared with control cells (0.99 ± 0.05 sec), (Fig. 2D). However, the half-lives in NuSAP^1–233^- (1.01 ± 0.06 sec) and NuSAP^delMCBD^-transfected cells (1.05 ± 0.07 sec) were similar to that of control cells (Fig. 2D). Because the turnover rate of MCAK relates to the stability of kinetochore microtubules, we further tested whether the stability of spindle microtubules could influence the dynamics of MCAK. We observed a reduced turnover rate of MCAK following treatment with 10 μM taxol treatment to stabilise microtubule plus ends (Fig. 2E,F). To determine whether the effect of NuSAP on microtubule stability was entirely dependent on its regulation of MCAK, we further examined the function of NuSAP in MCAK depleted-cells (Fig. S3B). Depletion of MCAK did not affect localisation of NuSAP (Fig. S3C), but the turnover rate of spindle microtubules was decreased when NuSAP was overexpressed in these cells (Fig. 2G,H), although this difference was not as marked as observed in the presence of MCAK (Fig. 1A,B). Taken together, this observation suggests that the NuSAP regulation of microtubule stability occurs predominantly through its effect on the dynamics of MCAK, but does not exclude further MCAK-independent mechanisms.

### NuSAP stabilises kinetochore microtubules through its regulation of MCAK depolymerisation activity

**Figure 3:**
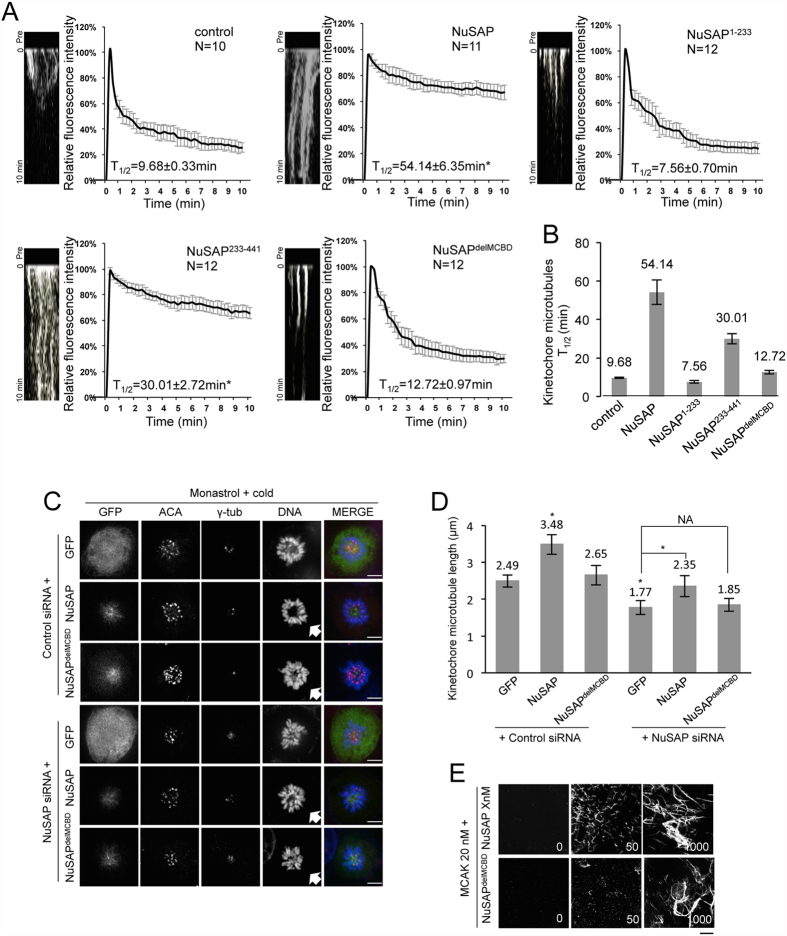
NuSAP stabilises kinetochore microtubules through its regulation on MCAK. (**A**) Representative kymographs and normalized signal recovery curves of photoactivation assays in metaphase HeLa cells expressing mCherry-vector (n = 12), mCherry-NuSAP (n = 12), mCherry-NuSAP^1–233^ (n = 14), mCherry-NuSAP^233–441^ (n = 13), or mCherry-NuSAP^delMCBD^ (n = 14). Solid lines represent the fit values of each group calculated by double exponential regression analysis, R^2^ > 0.99. (**B**) Bar chart representing the half-life of kinetochore microtubules calculated according to a double exponential regression curve. Error bars represent ± SD. (**C**) HeLa cells transfected with control siRNA or NuSAP siRNA together with GFP vector, GFP-NuSAP, or GFP-NuSAP^delMCBD^ were cold-treated with monastrol. Kinetochores were labelled with ACA, spindle poles with anti-γ-tubulin, and DNA with Hoechst 333342. Scale bar, 5 μm. (**D**) Bar chart represents average kinetochore microtubule length in HeLa cells transfected with control siRNA or NuSAP siRNA together with GFP vector, GFP-NuSAP, or GFP-NuSAP^delMCBD^ following cold-treatment with monastrol. Error bars represent ± SD. *p < 0.001. (**E**) Different concentrations of NuSAP or NuSAP^delMCBD^ and 20 nM MCAK proteins were incubated with 1.5 μM tubulin at 37 °C for 10 min. Scale bar, 20 μm.

To investigate whether the interaction between NuSAP and MCAK is essential to the regulation of kinetochore microtubule stability, we performed an FDAPA (fluorescence dissipation after photoactivation) experiment at the kinetochore region (Fig. S4A). A 405-nm laser was focused at a rectangular area (1 × 5 μm) around the metaphase plate, and the fluorescence intensity of the activated region was analysed and fitted to a double exponential decay curve (R^2^ > 0.99) with the slowly depolymerising populations corresponding to the kinetochore microtubules^39^. The kymographs and line-scan profiles indicated that the intensity of photoactivated PAGFP-α-tubulin was rapidly decayed in cells overexpressing NuSAP mutants lacking the MCBD, but was stable in those with an intact MCBD (Fig. 3A). Accordingly, the half-life of kinetochore microtubules was longer in NuSAP- and NuSAP^233–441^-overexpressing cells (54.14 ± 6.35 min and 30.01 ± 2.72 min, respectively) than in control cells (9.68 ± 0.33 min), consistent with previous reports^9^,^39^. The half-lives of kinetochore microtubules in NuSAP^1–233^- and NuSAP^delMCBD^-transfected cells (7.56 ± 0.70 min and 12.72 ± 0.97 min, respectively) was similar to that of control cells (Fig. 3A,B). Taken together, these results suggest that the interaction with MCAK is essential for NuSAP to stabilise kinetochore microtubules.

To confirm our results, we used super-resolution STED imaging to investigate the kinetochore microtubules more specifically (Fig. S4B). The line profiles shown in Fig. S4B (right panel) were generated from the enlarged images at a 25 nm resolution. We found that NuSAP and NuSAP^233–441^, containing an intact MCBD, but not NuSAP^1–233^ and NuSAP^delMCBD^, led to higher levels of stable kinetochore microtubule bundles after cold treatment, compared with the control. To rule out a possible dominant negative effect, we also examined kinetochore microtubule stability by depletion with NuSAP siRNA^11^ followed by rescue with NuSAP or NuSAP^delMCBD^ after monastrol-cold treatment (Fig. 3C,D). Kinetochore microtubule length was significantly increased in cells co-transfected with control siRNA and GFP-NuSAP (3.48 ± 0.27 μm) but not GFP-NuSAP^delMCBD^ (2.65 ± 0.26 μm), compared to the GFP vector control (2.49 ± 0.16 μm) (Fig. 3D, lane 1–3). Depletion of NuSAP significantly decreased the length of kinetochore microtubules (1.77 ± 0.19 μm) compared with control siRNA (lane 1 and 4). However, the kinetochore microtubule length was rescued in NuSAP-depleted cells co-transfected with GFP-NuSAP (2.35 ± 0.29 μm), but not GFP-NuSAP^delMCBD^ (1.85 ± 0.18 μm) (lane 5 and 6). To examine the regulation of NuSAP on MCAK depolymerisation activity *in vitro* further, a microtubule depolymerisation assay was performed with recombinantly expressed MCAK, NuSAP, and NuSAP^delMCBD^ proteins (Fig. S4D). The inhibitory effect of NuSAP on MCAK activity could be detected at a protein concentration of 50 nM. NuSAP^delMCBD^ did not inhibit depolymerisation (Fig. 3E), indicating that NuSAP specifically stabilises kinetochore microtubules through its regulation on the depolymerisation activity of MCAK during metaphase.

### Aurora B promotes the regulation of NuSAP on MCAK depolymerisation activity

**Figure 4:**
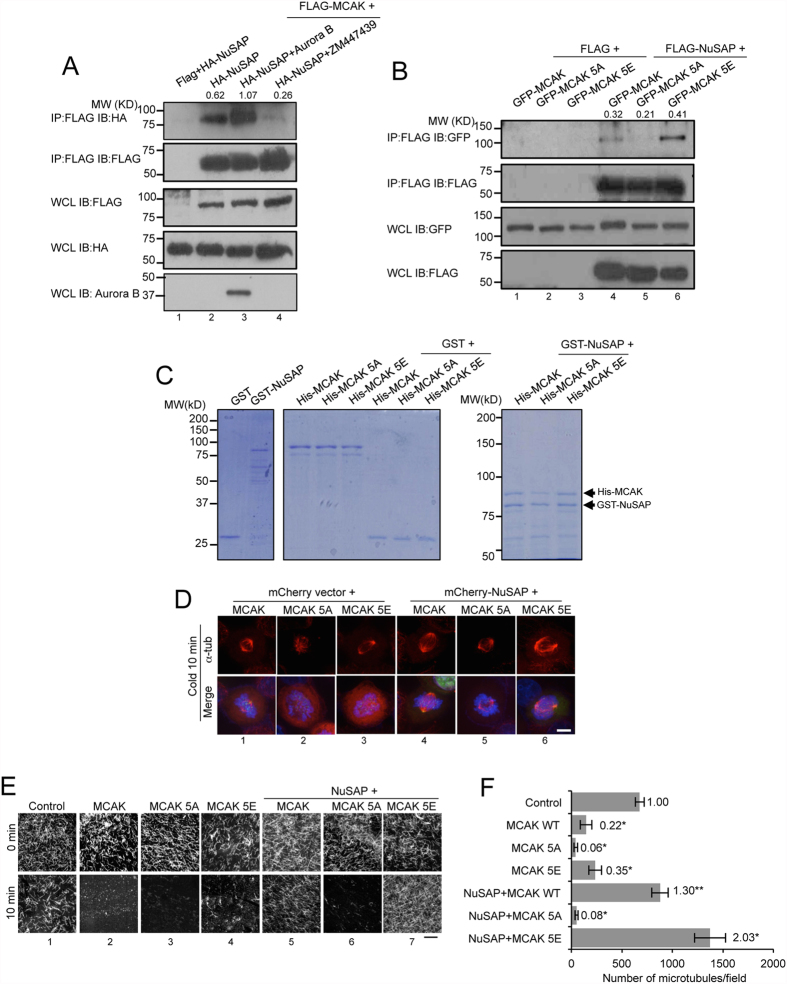
Aurora B positively regulates the function of NuSAP on MCAK depolymerisation activity. (**A**) Aurora B enhances the binding between NuSAP and MCAK. Whole cell lysates of 293T cells co-transfected with FLAG vector or FLAG-MCAK and HA-NuSAP in the presence of either Aurora B or 2 μM ZM447439 for 45 min were collected for co-immunoprecipitation (Co-IP) using FLAG-M2 beads. Immunoprecipitated proteins and whole cell lysates were detected with anti-HA and anti-FLAG antibodies. Quantification of the gel images with Image J indicates that the ratio of immunoprecipitated NuSAP to MCAK. (**B**) Whole cell lysates of HEK 293T cells co-transfected with a FLAG vector or FLAG-NuSAP and GFP-MCAK, GFP-MCAK 5A, or GFP-MCAK 5E were collected for Co-IP using FLAG M2-beads. Immunoprecipitated proteins and whole cell lysates were detected with anti-GFP and anti-FLAG antibodies. Quantification of the gel images with Image J indicates that the ratio of immunoprecipitated MCAK to NuSAP**. (C**) MCAK and MCAK 5E bind NuSAP *in vitro*. Purified His-MCAK, MCAK 5A, or MCAK 5E proteins were incubated with GST or GST-NuSAP and detected with Coomassie blue staining and western blotting in 10% (left panels) and 6% (right panel) SDS-PAGE. (**D**) Kinetochore microtubules in cold-treated metaphase HeLa cells expressing GFP-MCAK, GFP-MCAK 5A, or GFP-MCAK 5E with mCherry vector or mCherry-NuSAP. Cells were stained with anti-α-tubulin antibody and Hoechst 333342. Scale bar, 5 μm. (**E**) Microtubule depolymerisation assays with MCAK and NuSAP. NuSAP (100 nM) and 20 nM MCAK WT, MCAK 5A, or MCAK 5E proteins were incubated with 1.5 μM microtubules at 37 °C for 10 min. Scale bar, 20 μm. (**F**) Bar chart representing the number of microtubules in control, MCAK-, MCAK 5A-, and MCAK 5E-treated samples with or without NuSAP protein after a 10 min incubation. Three independent experiments were conducted. Error bars represent ± SD. *p < 0.001.

The depolymerisation activity of MCAK is tightly regulated by Aurora B kinase through its phosphorylation of MCAK at five serine residues (S92, S106, S108, S112, and S186)^40^. To determine the role of Aurora B kinase in regulating the interaction between NuSAP and MCAK, we performed immunoprecipitation assays using FLAG-tagged MCAK to pulldown HA-tagged NuSAP in HeLa cells. Strikingly, we observed that the association between NuSAP and MCAK was greatly enhanced by ectopically expressed Aurora B (Fig. 4A, lane 3), and that this protein-protein association was dramatically abolished by treatment with ZM447439, a specific inhibitor of Aurora B (Fig. 4A, lane 4), demonstrating that Aurora B positively regulates the interaction between MCAK and NuSAP. To further investigate whether the interaction between MCAK and NuSAP is dependent on Aurora B-mediated phosphorylation, we utilised phospho-deficient MCAK 5A and phospho-mimicking MCAK 5E^40^ mutants to verify the interaction between MCAK and NuSAP. As shown in Fig. 4B, the phospho-mimicking MCAK 5E mutant exhibited an enhanced ability to bind NuSAP, whereas the phospho-deficient MCAK 5A mutant displayed a considerably lower affinity to NuSAP. We further examined whether purified recombinant NuSAP could interact directly with purified recombinant MCAK and MCAK 5E *in vitro*. As shown in Fig. 4C, His-tagged recombinant MCAK and MCAK 5E were more efficiently pulled-down by GST-NuSAP compared with MCAK 5A. These results indicate that Aurora B is important in regulating the interaction of NuSAP and MCAK through phosphorylation of MCAK during metaphase.

To determine whether the depolymerisation activity of MCAK regulated by NuSAP is also dependent on Aurora B-mediated phosphorylation, we investigated kinetochore microtubule formation in MCAK-, MCAK 5A-, and MCAK 5E-transfected cells following cold treatment (Fig. 4D). Overexpression of NuSAP resulted in stable kinetochore microtubule formation in MCAK- and MCAK 5E-transfected cells (Fig. 4D, lane 4 and 6), but not MCAK 5A-transfected metaphase cells (Fig. 4D, lane 5). We also examined the depolymerising activity of purified recombinant MCAK, MCAK 5A, and MCAK 5E *in vitro* in taxol-stabilised microtubules in the presence of purified recombinant NuSAP^27^ (Fig. S4D). The presence of NuSAP restored microtubule depolymerisation induced by 20 nM MCAK and MCAK 5E (Fig. 4E, columns 5 and 7), but not by the phospho-deficient MCAK 5A (Fig. 4E, column 6). Quantification of this effect was performed by counting the number of microtubules per image, which confirmed the observation above (Fig. 4F). Taken together, these findings show that phosphorylation of MCAK by Aurora B plays a critical role in NuSAP-mediated MCAK depolymerisation activity.

## Discussion

During metaphase, multiple microtubule attachments are required at the kinetochores to correct defects in chromosome positioning and satisfy the spindle assembly checkpoint^41^. The dynamics of kinetochore microtubules are critical and must operate within a narrow permissible boundary to achieve stable kinetochore-microtubule attachment and accurate chromosome segregation^42^,^43^. Here, we identify NuSAP, a microtubule-associated protein, as a specific kinetochore microtubule stabiliser. Mechanistically, this is achieved through the regulation of MCAK, which functions by depolymerising microtubules. Thus, NuSAP regulates kinetochore microtubule dynamics by decreasing the turnover rate of kinetochore microtubules during metaphase. The temporal and spatial nature of the regulation indicates that NuSAP may regulate the length and stability of kinetochore microtubules in order to ensure correct chromosomal alignment at the metaphase plate.

We demonstrate that amino acids 433–441 at the C terminus of NuSAP are responsible for directly binding MCAK. The MCAK binding domain (MCBD) is essential for maintaining proper motility and depolymerisation activity of MCAK at the kinetochore region. MCAK is a robust microtubule depolymeriser^24^,^25^,^26^,^44^ and the dynamics of MCAK are tightly associated with correction of kinetochore-microtubule attachment 27,28,29,40, thus negative regulation by NuSAP is pivotal to the stabilisation of kinetochore microtubules once chromosomes are aligned during metaphase. Although several positive regulators of MCAK have been identified, e.g., inner centromere KinI stimulator (ICIS)^45^, TIP150^46^, and Kif18b^47^; the mechanism by which MCAK is negatively regulated was not well understood. Here, we reveal that NuSAP acts as a negative regulator of MCAK activity to alter the dynamics of kinetochore microtubules. To decipher the function of NuSAP on microtubule stability and its specific function on MCAK dynamics, the dynamics of MCAK on taxol stabilized microtubules is compared with the spindle microtubules in NuSAP overexpressing cells. Taken together the function of NuSAP on microtubule stability is deminished in MCAK depleted cells, our results show the important role of NuSAP on microtubule stability is majoraly through its regulation on MCAK. However, our current results do not exclude the possibility that NuSAP may act via additional MCAK-independent pathways to stabilise spindle microtubule formation. We show that NuSAP plays an indispensable role in ensuring precise MCAK function at kinetochore microtubules by tightly regulating the dynamics and depolymerisation activity of MCAK. Although NuSAP concentrates at the middle spindle region under the regulation of RanGTP, low amounts of NuSAP may also localise at the spindle pole region. Because MCAK localises at both ends of spindle poles, it would be interesting to study whether further interactions between MCAK and NuSAP occur at the spindle poles. Furthermore, our results also suggest that NuSAP may also play a more general role in spindle microtubule stability besides its function on kinetochore microtubules (Figs S2–4).

Aurora B plays a major role as a tension sensor in ensuring the proper dynamics of kinetochore microtubules and accurate kinetochore-microtubule attachment by regulating the phosphorylation of MCAK^27^,^29^,^40^,^48^. It has previously been demonstrated that MCAK mutants that are phospho-deficient (MCAK 5A) have increased depolymerisation activity, compared with the phospho-mimicking MCAK 5E mutant. Our data suggests that phosphorylation of MCAK by Aurora B is vital in determining its interaction with NuSAP, resulting in subsequent sequestration of its depolymerisation activity (Fig. 4). We propose that Aurora B acts as a molecular switch, intensifying the extra-temporal and spatial regulation of the NuSAP-MCAK machinery to ensure rapid changes in kinetochore microtubule dynamics and precise chromosome alignment during metaphase. Recent reports have indicated that Aurora B phosphorylation of MCAK induces a specific conformational switch directly related to its activity^49^,^50^. We observed that an inhibitor of Aurora B could indeed block the interaction between NuSAP and MCAK. However, the ability of phospho-deficient MCAK 5A to maintain binding to NuSAP, albeit at a lower level, suggests that MCAK may contain further Aurora B phosphorylation sites that regulate its conformation. Some reports have shown that Aurora B can diffuse from the centrosome across a gradient to phosphorylate other mitotic proteins^51^,^52^ and that NuSAP itself can be phosphorylated^53^,^54^,^55^. We therefore cannot rule out that, in addition to Aurora B, other kinases may also regulate the phosphorylation of NuSAP or MCAK, which in turn regulate the kinetochore microtubule dynamics.

Based on our results, we propose a model to represent the role of NuSAP in stabilising kinetochore microtubules through negative regulation of MCAK depolymerising activity (Fig. S5). During metaphase, MCAK diffuses into the kinetochore region to depolymerise kinetochore microtubules and maintain appropriate kinetochore microtubule dynamics. The strong interaction between NuSAP and MCAK results in localisation of MCAK into the Aurora B activated region^33^,^34^,^56^ where it can be phosphorylated by Aurora B, which further enhances the interaction between MCAK and NuSAP. This results in a marked reduction in the depolymerisation activity of MCAK, which in turn promotes kinetochore microtubule stability.

Our study provides new insights into the mechanism by which NuSAP is able to stabilise kinetochore microtubules to ensure accurate chromosome alignment and spindle assembly during mitosis and aids our understanding of how NuSAP deficiencies might lead to the presence of mitotic defects.

## Materials and Methods

### Plasmid construction and siRNA

NuSAP and MCAK cDNAs were amplified from a cDNA library extracted from HEK 293T cells, and inserted into the pXJ40 vector tagged with GFP, mCherry, HA, FLAG, or PAGFP. The truncated mutants of NuSAP were constructed with the use of PfuTurbo^TM^ polymerase (Stratagene). The mammalian cell expression plasmids of GFP-MCAK-His, GFP-MCAK 5A-His, and GFP-MCAK 5E-His mutants were obtained from Addgene (#13987, #23108, #23109^40^; contributed by Dr Linda Wordeman’s group). The sequences of NuSAP siRNA (5′-AAGCACCAAGAAGCTGAGAAT-3′^11^) and MCAK siRNA (5′-GCAATAAACCCAGAACTCT-3′^47^) were described previously and synthesized by Sigma. Silencer Negative Control #1 siRNA was used as a negative control (Ambion).

### Cell culture, transfection, and synchronisation

HeLa and HEK 293T cells (ATCC) were cultured in Dulbecco’s modified Eagle medium (DMEM, Sigma) supplemented with 10% foetal bovine serum (Gibco) and 1% penicillin/streptomycin at 37 °C with 5% CO_2_. HEK 293T and HeLa cells were transfected using either calcium phosphate or Effectene^TM^ (Qiagen) to introduce target genes. For siRNA transfections, HeLa cells were transfected at 30–50% confluence with 20 nM of each siRNA for 48 h using Lipofectamine 2000 (Invitrogen) according to the manufacturer’s protocol. To synchronise cells at the G2/M stage, HeLa cells were treated with 100 ng/ml nocodazole for 16 h and washed three times with 1 × PBS and then released into DMEM medium with 10 μm MG132 (Sigma) for 2 h.

### Protein expression and pull-down assay

His-NuSAP and His-NuSAP^delMCBD^ were inserted into the pET28b vector. GST-NuSAP was constructed in the pGEX-4T-1 vector. GST-NuSAP, His-NuSAP, and His-NuSAP^delMCBD^ were expressed in *E. coli* BL21 (DE3) pLysS at 16 °C overnight following IPTG induction. Proteins were purified with glutathione-Sepharose 4B (Amersham Biosciences) or Ni-NTA agarose (Qiagen), as appropriate. His-MCAK, 5A, and 5E were inserted into the pFastBac Dual vector, transfected into SF9 cells using Cellfectin^TM^ (Invitrogen) and expressed at 28 °C for three days. MCAK, MCAK 5A, and MCAK 5E were expressed and purified as described in previous studies 24,47. *In vitro* GST pull-down assays with purified proteins were performed with 3 μg GST fusion protein and 2 μg target protein in 50 mM Tris-HCl, pH 7.4, 150 mM NaCl, 2 mM EGTA, and 0.5% Triton X-100 46. After overnight incubation at 4 °C, the GST beads were washed three times and protein samples were separated by 10% or 6% SDS-PAGE as indicated and analysed by Coomassie Blue staining.

### Mass Spectrometry

To identify possible NuSAP binding partners, anti-FLAG-M2 agarose beads (Sigma) were incubated with FLAG-NuSAP- or FLAG vector-expressing HEK 293T cell lysates for 3 h at 4 °C. The beads were washed five times in mammalian cell lysis buffer (50 mM HEPES, 100 mM NaCl_2_, 1 mM EDTA, 1% Triton X-100, and 10% glycerol), and bound species were separated on a 10% SDS-PAGE gel stained with Coomassie blue. The selected bands were analysed with a Triple TOF 5600 mass spectrometer (ABSciex), and the data were analysed with ProteinPilot 4.0 (ABSciex).

### Immunoprecipitation and western blot

For immunoprecipitation assays, HEK 293T cells were lysed in M-PER^TM^ (Thermo Scientific) with 1 mM Na_3_VO_4_, 10 μg/ml aprotinin, 1 mM pepstatin, 1 mM leupeptin, and 1 mM PMSF. Nocodazole (10 μg/ml) was added to depolymerise microtubules. FLAG M2 beads (10 μl) (Sigma) were incubated with cell lysates for 1 h at 4 °C and washed three to five times in mammalian cell lysis buffer. Protein samples were separated on a 10% SDS gel and detected by primary antibodies (rabbit anti-FLAG, rabbit anti-HA, rabbit anti-GFP (Sigma), rabbit anti-Aurora B (Cell Signalling), rabbit anti-NuSAP (Abcam), mouse anti-importin-β (Abcam)) followed by horseradish peroxidase (HRP)-conjugated secondary antibodies (Santa Cruz Biotechnology).

### Immunofluorescence and microtubule regrowth assay

HeLa cells were cultured on ethanol-sterilised coverslips in 12-well plates and synchronised prior to fixation with ice-cold methanol at −20 °C for 10 min. Fixed cells were permeabilised with 0.3% Triton X-100 in 1 × PBS for 15 min and blocked with 2% BSA for 30 min at room temperature. Mouse anti-α-tubulin 1:2000 (Sigma), mouse anti-acetylated α-tubulin 1:200 (Sigma), rabbit anti-γ-tubulin 1:200 (Sigma), and rabbit anti-MCAK (Cytoskeleton) were used to stain the respective proteins. DNA was stained with Hoechst 33342 (Invitrogen). Confocal images were collected using the UltraVIEW Vox Spinning disc confocal system (PerkinElmer). Images were processed using Volocity^TM^ software (PerkinElmer) or Image J (National Institutes of Health, Bethesda, MD). STED imaging was conducted with the STED TCS SP8 system (Leica Microsystems) and both the STED and confocal images were deconvoluted using Huygens Titan (Scientific Volume Imaging). For microtubule regrowth assays, synchronized HeLa cells were washed with cold medium and placed on ice for 30 min before changing into prewarmed medium. The cells were fixed and immunostained at the indicated time points after medium replacement to monitor microtubule regrowth^57^.

### Live-cell imaging

HeLa cells were cultured on 35-mm glass-bottom petri dishes (Greiner Bio-One), and imaging was conducted at 37 °C. To depolymerise tubules, a final concentration of 10 μm nocodazole was added to the cultured cells, and images were acquired at 10-sec intervals for 3 min. The images were obtained with an UltraVIEW Vox Spinning disc confocal system (PerkinElmer), an Olympus Uplan SApo 100 × 1.4 oil lens, and EMCCD camera C9100-50 (Hamamatsu). The images were processed using either Volocity^TM^ software (PerkinElmer) or Image J (National Institutes of Health, Bethesda, MD), as required.

For FLIP experiments, synchronised mCherry-α-tubulin stable HeLa cells were cultured on glass-bottom dishes and imaged with an UltraVIEW Vox spinning disc confocal system (PerkinElmer) in a 37 °C humid chamber supplied with 5% CO_2_. A photobleaching laser (405 nm, 50 mW) was utilised at two spots (2 × 2 μm) away from the mitotic spindle. Images of 20 mitotic cells per transfection were acquired at 5-sec intervals for 3 min. The 568-nm fluorescence signal intensity was background-corrected and normalised to 100% at the first time point by means of Volocity^TM^ software. The turnover half-time of mCherry α-tubulin was calculated by linear regression as previously described^58^.

For FRAP experiments, GFP-MCAK-expressing HeLa cells were synchronised at the metaphase stage, cultured in a 37 °C humid chamber supplied with 5% CO_2,_ and imaged with the use of an UltraVIEW Vox spinning disc confocal system (PerkinElmer). A 1 × 1-μm photobleaching spot was placed at the kinetochore region. GFP fluorescence intensities were photobleaching- and background-corrected and analysed with Volocity^TM^ 3D imaging analysis software.

For photoactivation experiments, PAGFP-α-tubulin-expressing HeLa cells were synchronised and labelled with Hoechst 33342 (2.5 ng/ml, 10 min) before photoactivation. A 405-nm laser at 15% intensity was focused on the selected area for 1 sec, and images were acquired at 15-sec intervals for 10 min. Kymographs were analysed with Image J. Fluorescence intensity of the activated region was photobleaching- and background-corrected with Volocity^TM^ and analysed by double exponential regression analysis with Sigmaplot software (Jandel Scientific) to fit the data to the equation F(t) = A_1_e^−k1t^ + A_2_e^−k2t^, where F(t) is the fluorescence intensity over time, A_1_ and A_2_ represent the proportions of interpolar microtubules and kinetochore microtubules with regression rates k_1_ and k_2_, respectively. Kinetochore microtubule turnover half-life was calculated using the equation T_1/2_ = Ln2/k_2_^39^,^59^.

### *In vitro* microtubule assays and electron microscopy

For *in vitro* microtubule stabilisation assays, a final concentration of 20 μM tubulin (Cytoskeleton) in BRB80 buffer (80 mM PIPES, pH 6.9; 1 mM MgCl_2_; 1 mM EGTA) containing 1mM GTP was incubated at 37 °C for 10 min with the indicated amounts of NuSAP^13^. To form GMPCPP microtubules, tubulin was incubated with 1 mM GMPCPP at 37 °C for 30 min^60^. One micromolar NuSAP protein was incubated with the GMPCPP microtubules for 10 min. For *in vitro* microtubule destabilisation assays, tubulins were first polymerised in BRB80 buffer containing 1 mM GTP at 37 °C for 15 min. Varying concentrations of NuSAP or NuSAP^delMCBD^ were pre-incubated with 20 nM MCAK, MCAK 5A, or MCAK 5E in BRB80 buffer containing 1 mM ATP at room temperature for 5 min and then added to a reaction mixture containing 1.5 μM polymerised microtubules with 10 μM taxol^27^. After a further 10 min, the microtubule samples were fixed on a cover glass in ice-cold methanol at −20 °C for 5 min and stained with mouse anti-α-tubulin 1:2000 (Sigma). Images were obtained with an Axio Imager II system and Zeiss 63 × 1.4 oil lens (Zeiss), and the numbers of microtubules were analysed by ImageJ (National Institutes of Health, Bethesda, MD). For electron microscopy, microtubules (1.5 μM) were polymerised with 10 μM taxol and incubated alone or with 1 μM NuSAP protein for 10 min. The samples were spotted on holey-carbon film and fixed with UA for electron microscopy. Images were taken on a Joel 2010F electron microscope^61^,^62^.

## Additional Information

**How to cite this article**: Li, C. *et al.* NuSAP modulates the dynamics of kinetochore microtubules by attenuating MCAK depolymerisation activity. *Sci. Rep.* **6**, 18773; doi: 10.1038/srep18773 (2016).

## Supplementary Material

Supplementary Information
