# Supplementary material for: NuSAP modulates the dynamics of kinetochore microtubules by attenuating MCAK depolymerisation activity
Source: Sci Rep. 2016 Jan 6;6:18773. doi: 10.1038/srep18773 (PMC4702128; doi:10.1038/srep18773)
Supplement: Supplementary Information [file srep18773-s1.pdf]

Supplementary Information

NuSAP modulates the dynamics of kinetochore microtubules by attenuating MCAK depolymerisation activity

Chenyu Li[1,4], Yajun Zhang[1], Qiaoyun Yang[1], Fan Ye[1], Stella Ying Sun[1], Ee Sin Chen[2], Yih-Cherng Liou[1,3,*]

[1]Department of Biological Sciences, Faculty of Science, National University of Singapore, 14 Science Drive 4, 117543, Republic of Singapore
[2]Department of Biochemistry, Yong Loo Lin School of Medicine, National University of Singapore, Singapore 117597, Republic of Singapore.
[3]NUS Graduate School for Integrative Sciences and Engineering, National University of Singapore, Singapore 117573, Republic of Singapore
[4]Present address: Harvard Medical School, Center for Life Science 0428, Beth Israel Deaconess Medical Center, Boston, Massachusetts 02215, USA

* Correspondence and requests for materials should be addressed to Y.-C.L. (Email: dbslyc@nus.edu.sg)

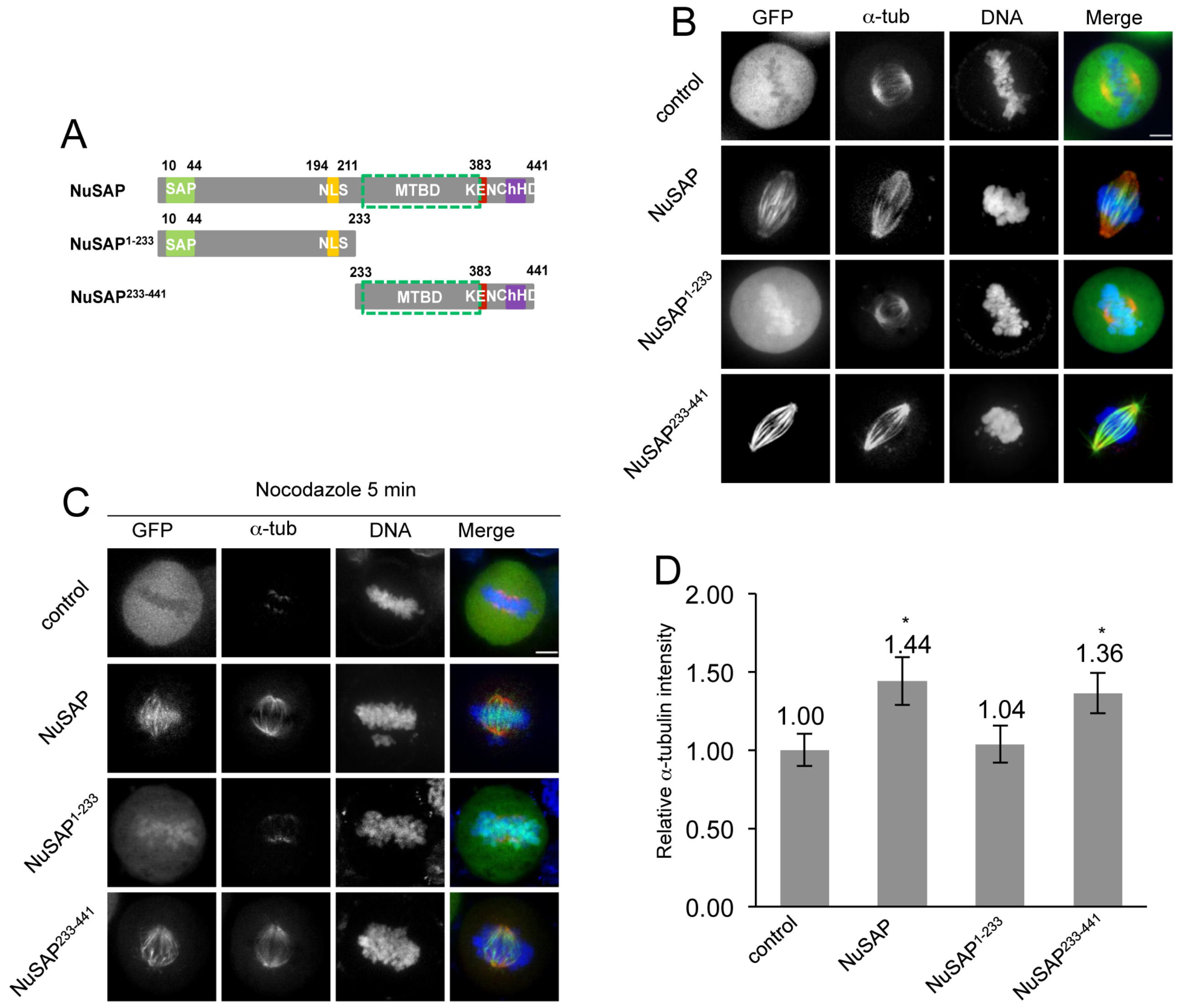

Figure S1. NuSAP stabilises spindle microtubules during metaphase.
(A) A schematic diagram of NuSAP and its truncation mutants $NuSAP^{1\text{-}233}$, $NuSAP^{233\text{-}441}$. Previously identified functional domains on NuSAP are indicated. SAP: 10-44 aa, chromosome binding domain; NLS domain: 194-211 aa, Nuclear Localization Signal; predicted microtubule binding domain (dotted line square): 233-383 aa; KEN box: 384-390 aa, APC/C binding domain; ChHD domain: 410-433 aa, Charged Helical Domain (Iyer et al, 2011). (B) Fluorescent images of metaphase HeLa cells expressing GFP-vector (control), GFP-NuSAP, GFP-$NuSAP^{1\text{-}233}$ or GFP-$NuSAP^{233\text{-}441}$. Mitotic spindles were labeled with an anti-α-tubulin antibody and DNA with Hoechst 333342. Scale bar, 5μm. (C) Stable spindle microtubules in metaphase HeLa cells expressing GFP-vector (control), GFP-NuSAP, GFP-$NuSAP^{1\text{-}233}$ and GFP-$NuSAP^{233\text{-}441}$ after nocodazole treatment (10μM, 5min). Mitotic spindles were labeled with an anti-α-tubulin antibody and DNA with Hoechst 333342. Scale bar, 5μm. (D) Bar chart representing the average of α-tubulin immunofluorescence intensity on metaphase spindles stained as in C in cells expressing GFP-NuSAP, GFP-$NuSAP^{1\text{-}233}$, GFP-$NuSAP^{233\text{-}441}$ and GFP-vector only (control). The number of cells quantified: n (GFP)=40/3 independent experiments, n (GFP-NuSAP)=41/3, n (GFP-$NuSAP^{1\text{-}233}$)=39/3, n (GFP-$NuSAP^{233\text{-}441}$)=36/3. Error bars represent ±SD. * $p<0.001$.

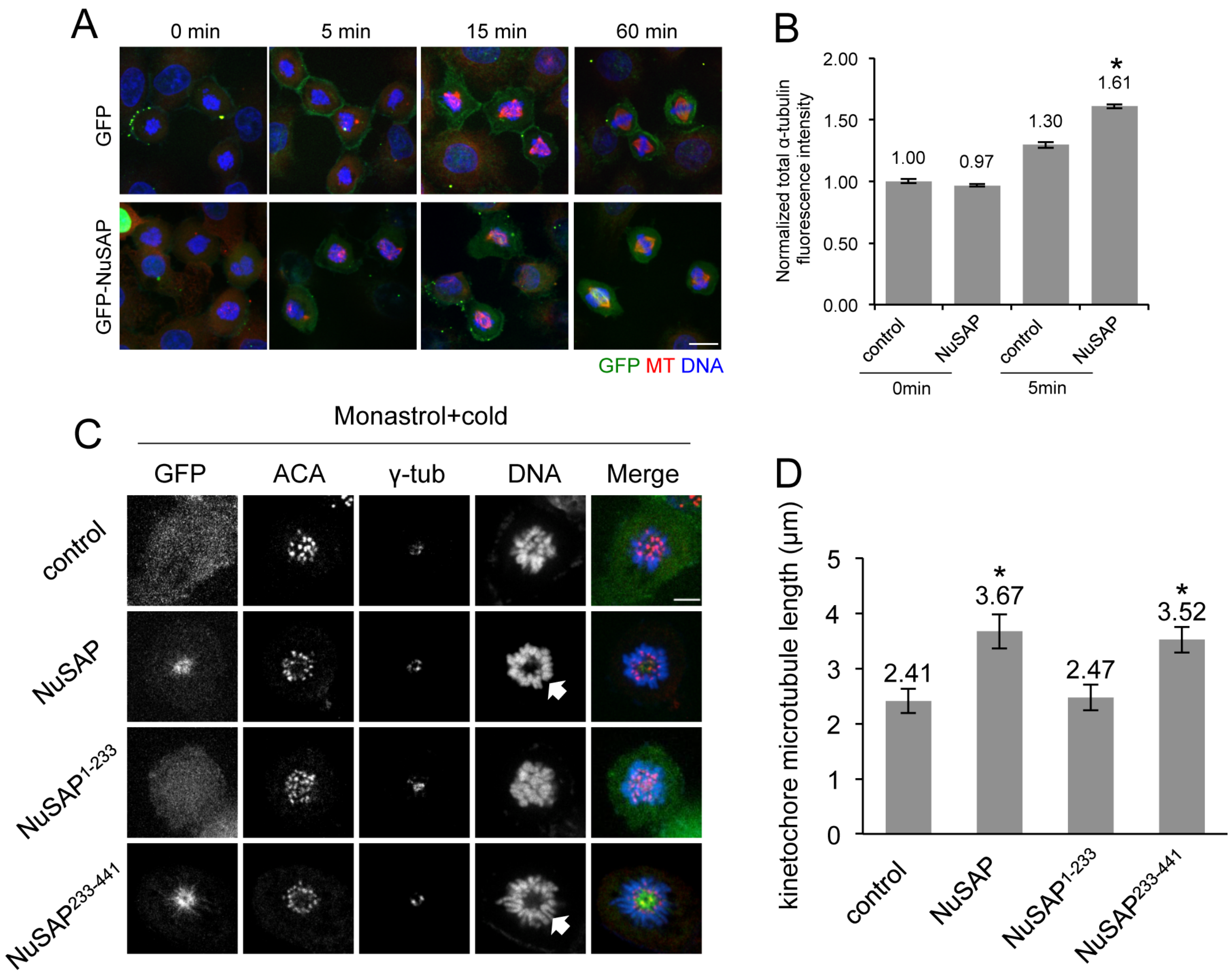

Figure S2. NuSAP bundles microtubules *in vitro* and enhances tubulin assembly.
(A) GFP vector or GFP-NuSAP transfected synchronized HeLa cells were placed on ice for 30 min and microtubule regrowth was induced with prewarmed medium for indicated time points. Scale bar, 10μm. (B) The total α-tubulin fluorescence intensity was quantified. n=20 cells/3 independent experiments. Error bars, SD. *p<0.001. (C) NuSAP and NuSAP$^{233\text{-}441}$ stabilise kinetochore microtubules, but not NuSAP$^{1\text{-}233}$. HeLa cells expressing GFP-NuSAP, GFP-NuSAP$^{1\text{-}233}$, GFP-NuSAP$^{233\text{-}441}$, or GFP-vector (control) were cold-treated with monastrol. Kinetochores were labelled with ACA, spindle poles with anti-γ-tubulin, and DNA with Hoechst 333342. Scale bar, 5 μm. (D) Bar chart representing average kinetochore microtubule length in HeLa cells expressing GFP-NuSAP, GFP-NuSAP$^{1\text{-}233}$, GFP-NuSAP$^{233\text{-}441}$, or GFP-vector after cold treatment with monastrol. Error bars represent ± SD. * p < 0.001.

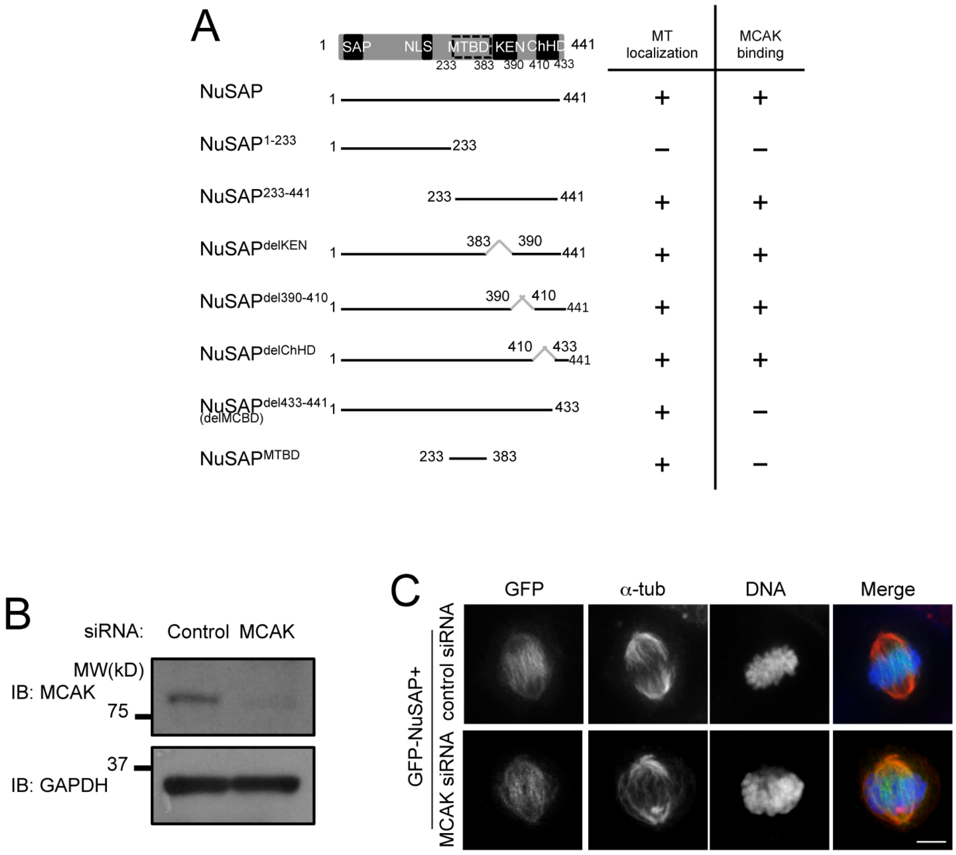

Figure S3. NuSAP interacts with MCAK through its MCBD.
(A) A schematic map of NuSAP and its truncation mutants. The table represents microtubule localization and MCAK binding ability of different truncation mutants. +, positive; -, negative. (B) The effectiveness of MCAK depletion in HeLa cells was analyzed by western blot 48 hr after siRNA treatment. (C) Fluorescent images of GFP-NuSAP in control siRNA or MCAK siRNA transfected HeLa cells. Mitotic spindles were labeled with an anti-α-tubulin antibody and DNA with Hoechst 333342. Scale bar, 5μm.

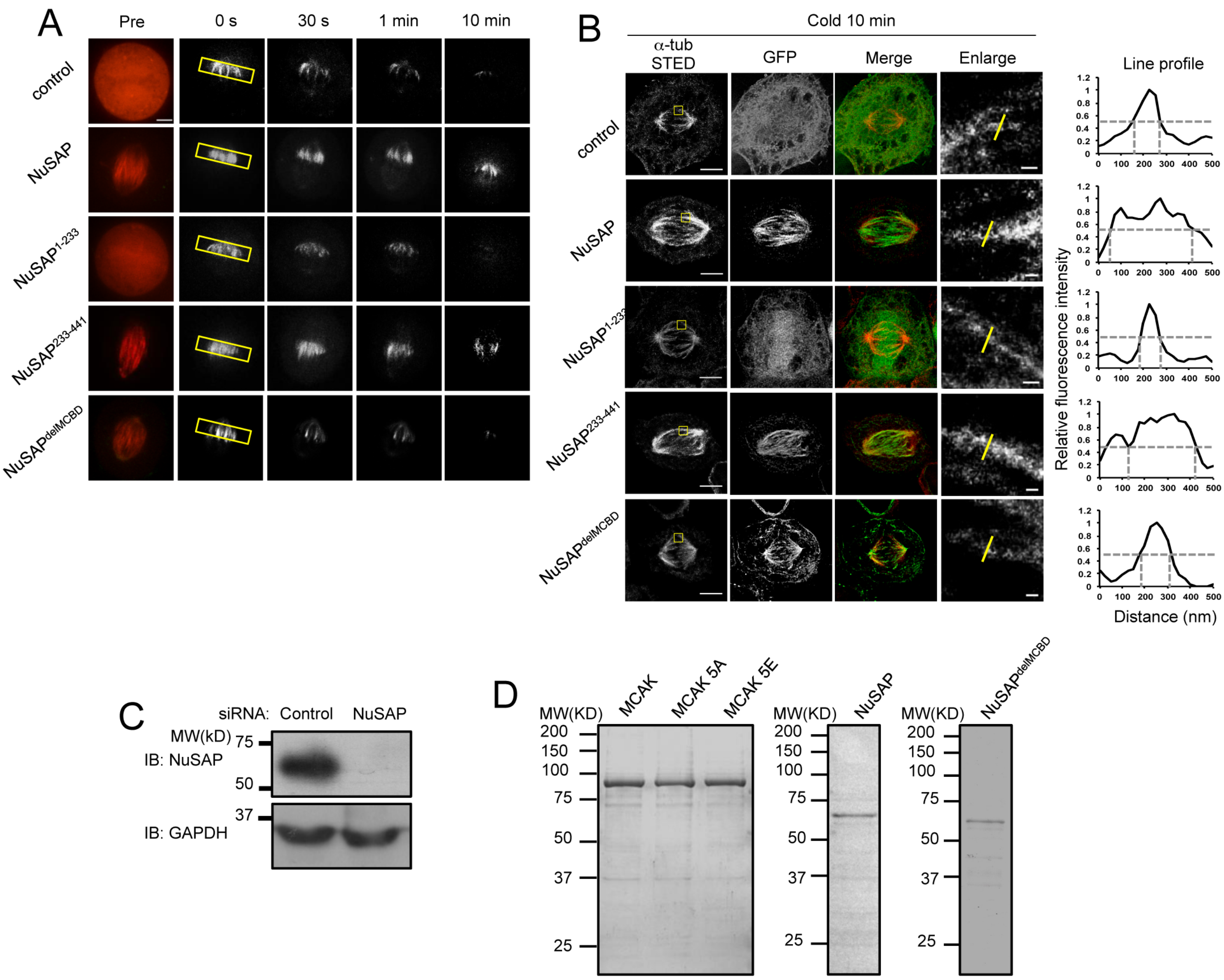

Figure S4. NuSAP stabilises kinetochore microtubules through its negative regulation of MCAK.

(A) Representative images of PAGFP α-tubulin stability at the kinetochore region in metaphase HeLa cells expressing mCherry-NuSAP, mCherry-NuSAP$^{1\text{-}233}$, mCherry-NuSAP$^{233\text{-}441}$, mCherry-NuSAP^{delMCBD} or mCherry-vector (control) after photoactivation. Yellow squares represent photoactived region. Images were acquired in 15-sec intervals for 10 min. Scale bar, 5μm. (B) NuSAP and NuSAP$^{233\text{-}441}$ stabilise kinetochore microtubules, but not NuSAP^{delMCBD}. HeLa cells expressing GFP-NuSAP, GFP-NuSAP$^{1\text{-}233}$, GFP-NuSAP$^{233\text{-}441}$, GFP-NuSAP^{delMCBD} or GFP-vector (control) were treated with a cold method to remove interpolar microtubules. Kinetochore microtubules were labeled with anti-α-tubulin and imaged with STED method (Pixel size 25nm). Scale bar, 5μm. The indicated regions were enlarged and analyzed with line profile. Scale bar, 300nm. (C) The effectiveness of NuSAP depletion in HeLa cells was analyzed by western blot 48 h after siRNA treatment. (D) Purification of His-tagged NuSAP and NuSAP^{delMCBD} protein from bacteria and His-tagged MCAK WT, MCAK 5A, and MCAK 5E from sf9 cells. A total of 2.5μg of NuSAP, NuSAP^{delMCBD}, MCAK WT, MCAK 5A and MCAK 5E proteins were stained using Coomassie blue staining, respectively.

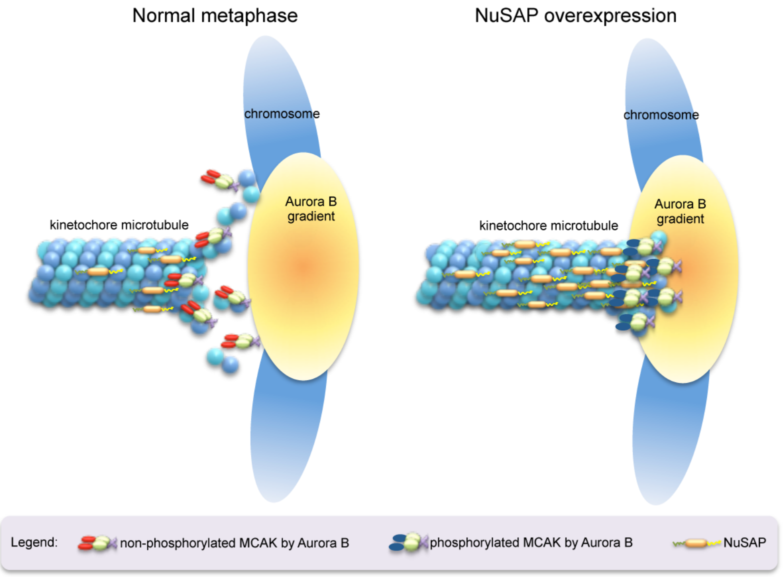

Figure S5. Aurora B regulates the NuSAP function on MCAK depolymerisation activity.
The schematic model represents the role of NuSAP in stabilising kinetochore microtubules through regulating MCAK, which is regulated by Aurora B kinase. In metaphase cells, NuSAP interacts with MCAK at kinetochore microtubules to maintain proper kinetochore microtubule dynamics. In NuSAP-overexpressing cells, the interaction of NuSAP with MCAK results in localisation of MCAK in the Aurora B region where it is phosphorylated, leading to further NuSAP-MCAK interactions, and a reduction in MCAK depolymerising activity.

Table S1. The identified peptides of NuSAP, Importin β and MCAK by mass spectrometry analyses.

Accession	Name	Peptides(95%)	Unused	%Cov(95)
sp|Q14974| IMB1_HUMAN	Importin subunit beta-1 OS=Homo sapiens GN=KPNB1 PE=1 SV=2	41	62.48	43.48999858
	Protein sequence coverage:<br>**MELITILEKTVSPDRLELEAAQK**FLER**AAVENLPTFLVELSRVLANPGNSQVAR**VAAGLQIKNSLT<br>SKDPDIKAQYQQR**WLAIDANAR**REVK**NYVLQTLGTETYR**PSSASQCVAGIACAEIPVNQWPELIP<br>QLVANVTNPNSTEHMKESTLEAIGYICQDIDPEQLQDK**SNEILTAIIQGMR**KEEPSNNVK**LAATNAL**<br>**LNSLEFTK**ANFDKESER**HFIMQVVCEATQCPDTR**VR**VAALQNLVK**IMSLYYQYMETYMGPALFAI<br>TIEAMKSDIDEVALQGIEFWSNVCDEEMDLAIEASEAAEQGRPPEHTSKFYAK**GALQYLVPILTQT**<br>**LTKQDENDDDDDWNPCK**AAGVCLMLLATCCEDDIVPHVLPFIKEHIKNPDWRYRDAAVMAFGCIL<br>EGPEPSQLKPL**VIQAMPTLIELMK**DPSVVVR**DTAAWTVGR**ICELLPEAAINDVYLAPLLQCLIEGLS<br>AEPR**VASNVCWAF**SSLAEAAYEAADVADDQEEPATYCLSSSFELIVQK**LLETTDRPDGHQNNLR**<br>**SSAYESLMEIVK**NSAKDCYPAVQK**TTLVIMERLQQVLQMESHIQSTSDR**IQFNDLQSLLCATLQNV<br>LRK**VQHQDALQISDVVMASLLR**MFQSTAGSGGVQEDALMAVSTLVEVLGGEFLK**YMEAFKPFL**<br>**GIGLK**NYAEYQVC**LAAVGLVGDLCR**ALQSNIIPFCDEVMQLLLENLGNENVHRSVKPQILSVFGDI<br>ALAIGGEFKK**YLEVVLNTLQQASQAQVDKSDYDMVDYLNELRESCLEAYTGIVQGLKGDQENV**<br>**HPDVMLVQPR**VEFILSFIDHIAGDEDHTDGVVAC**AAGLIGDLCTAFGK**DVLKLVEAR**PMIHELLTE**<br>**GR**RSKTNKAK**TLATWATK**ELRKLKNQA	AAGLIGDLCTAFGK<br>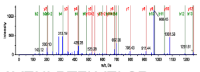<br>AAVENLPTFLVELSR<br>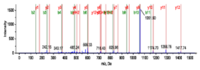<br>DTAAWTVGR<br>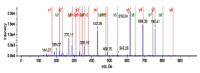	ESCLEAYTGIVQGLK<br>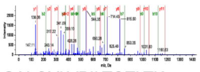<br>GALQYLVPILTQTLTK<br>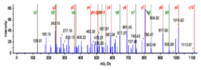<br>HFIMQVVCEATQCPDTR<br>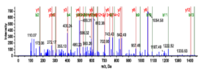	
sp|Q9BXS6| NUSAP_HUMAN	Nucleolar and spindle-associated protein 1 OS=Homo sapiens GN=NUSAP1 PE=1 SV=1	5	9.77	14.74000067
	Protein sequence coverage:<br>MIIPSLEELDSLK**YSDLQNLAK**SLGLRANLRATKLLKALKGYIKHEARKGNENQDESQTSASSCDE<br>TEIQISNQEEAERQPLGHVTKTRRRCKTVRVDPDSQQNHSEIK**ISNPTEFQNHEK**QESQDLRATA<br>K**VPSPPDEHQEAENAVSSGNR**DSKVPSEGKKSLYTDESSKPGKNKRTAITTPNFKKLHEAHFKE<br>MESIDQYIERKKK**HFEEHNSMNELK**QQPINKGGVRTPVPPRGR**LSVASTPISQR**RSQGRSCGPA<br>SQSTLGLKGSLKRSAISAAKTGVRFSAATKDNEHKRSLTKTPARKSAHVTVSGGTPKGEAVLGTH<br>KLKTITGNSAAVITPFK**LTTEATQTPVSNK**KPVFDLKASLSRPLNYEPHKGKLKPWGGQSKENNYLN<br>QHVNRINFYKKTYKQPHLQTKEEQRKKREQERKEKKAKVLGMRRGLILAED	LTTEATQTPVSNK<br>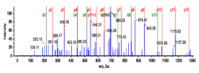<br>CPSPPDEHQEAENAVSSGNR<br>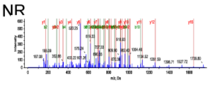<br>YSDLQNLAK<br>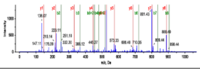	ISNPTEFQNHEK<br>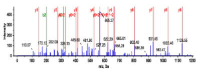<br>LSVASTPISQR<br>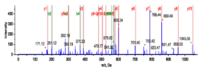	
sp|Q99661| KIF2C_HUMAN	Kinesin-like protein KIF2C OS=Homo sapiens GN=KIF2C PE=1 SV=2	2	3.72	2.483000048
	Protein sequence coverage:<br>MAMDSSLQARLFPGLAIKIQRSNGLIHSANVRTVNLEKSCVSVEWAEGGATKGKEIDFDDVAAINP<br>ELLQLLPLHPKDNLPLQENVTIQKQKRRSVNSKIPAPKESLRSRSTRMSTVSELRITAQENDMEVE<br>LPAAANSRKQFSVPPAPTRPSCPAVAEIPLRMVSEEMEEQVHSIRGSSSANPVNSVRRKSCLVKE<br>VEKMKNKREEKKAQNSEMRMKRAQEYDSSFPNWEFARMIKEFRATLECHPLTMTDPIEEHRICV<br>CVRKRPLNKQELAKKEIDVISIPSKCLLLVHEPKLKVDLTKYLENQAFCFDFAFDETASNEVVYRFT<br>ARPLVQTIFEGGKATCFAYGQTGSGKTHTMGGDLSGKAQNASKGIYAMASRDVFLLKNQPCYRK<br>LGLEVYVTFFEIYNGK**LFDLLNK**KAKLRVLEDGKQQVQVVGLQEHLVNSADDVIKMIDMGSACRT<br>SGQTFANSNSSRSHACFQIILRAKGRMHGK**FSLVDLAGNER**GADTSSADRQTRMEGAEINKSLL<br>ALKECIRALGQNKAHTPFRESKLTQVLRDSFIGENSRTCMIATISPGISSCEYTLNTLRYADRVKEL<br>SPHSGPSGEQLIQMETEEMEACSNGALIPGNLSKEEEELSSQMSSFNEAMTQIRELEEKAMEEL<br>KEIIQQGPDWLELSEMTEQPDYDLETFVNKAESALAQQAKHFSALRDVIKALRLAMQLEEQASRQ<br>ISSKKRPQ	FSLVDLAGNER<br>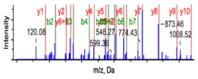	LFDLLNK<br>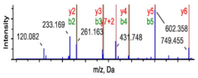	

ProteinPilot, confidence score: 95%
